# How do Senior Center Attendees Rate the Importance of Programs and Services Compared to Non-Attendees?

**DOI:** 10.1093/geroni/igaa057.383

**Published:** 2020-12-16

**Authors:** Ceara Somerville, Saralyn Collins, Shayna Gleason, Caitlin Coyle

**Affiliations:** 1 University of Massachusetts Boston, Everett, Massachusetts, United States; 2 University of Massachusetts Boston, Boston, Massachusetts, United States

## Abstract

Senior centers provide a wide range of programs and services to meet the needs of the growing aging population. As senior centers aim to serve all older community-members, it is important to assess the value of these services through the lens of both attendees and non-attendees of senior centers. Using a sample of 4,750 community-dwelling adults age 60 or older from Massachusetts, this project aims to analyze perceptions of program importance by center attendees versus non-attendees. Almost 60% of the sample never attend a senior center. Nearly a third of center attendees ranked exercise classes and education opportunities as important, compared to about 20% of non-attendees. Almost half of attendees rated application assistance and nutrition programs as not important, compared to 38% and 40% of non-attendees, respectively. Conversely, for all programs listed, non-users more frequently checked “unsure” of program/service importance. This was especially true for exercise classes, professional services, social or recreational activities, educational opportunities, and trips, for which 18% of non-attendees selected unsure. Center attendees more frequently view the programs/services offered at the senior center as important or very important, likely because they have greater awareness. Results highlight that that non-attendees are unsure of the value of programs or have no interest the programs and services provided. Not only do these results provide an opportunity to senior centers to assess how valuable certain programs and services are, they also emphasize the need for continued information streams regarding programs and services offered at senior centers to those who are unfamiliar.

